# Interferon Lambda 3/4 (IFNλ3/4) rs12979860 Polymorphisms Is Not Associated With Susceptibility to Systemic Lupus Erythematosus, Although It Regulates OASL Expression in Patients With SLE

**DOI:** 10.3389/fgene.2021.647487

**Published:** 2021-06-02

**Authors:** Yaneli Juárez-Vicuña, Julia Pérez-Ramos, Laura Adalid-Peralta, Fausto Sánchez, Laura Aline Martínez-Martínez, María del Carmen Ortiz-Segura, Edgar Pichardo-Ontiveros, Adrián Hernández-Díazcouder, Luis M. Amezcua-Guerra, Julian Ramírez-Bello, Fausto Sánchez-Muñoz

**Affiliations:** ^1^Department of Immunology, Instituto Nacional de Cardiología Ignacio Chávez, Mexico City, Mexico; ^2^Department of Biological Systems, Universidad Autónoma Metropolitana-Xochimilco, Mexico City, Mexico; ^3^Unit for the Study of Neuroinflammation in Neurological Pathologies, Instituto de Investigaciones Biomédicas, Instituto Nacional de Neurología y Neurocirugía, Mexico City, Mexico; ^4^Department of Agricultural and Animal Production, Universidad Autónoma Metropolitana-Xochimilco, Mexico City, Mexico; ^5^Department of Rheumatology, Instituto Nacional de Cardiología Ignacio Chávez, Mexico City, Mexico; ^6^Laboratory of Pharmacology and Toxicology, Hospital Infantil de México Federico Gómez, Mexico City, Mexico; ^7^Department of Nutrition Physiology, Instituto Nacional de Ciencias Médicas y Nutrición Salvador Zubirán, Mexico City, Mexico; ^8^Department of Health Care, Universidad Autónoma Metropolitana-Xochimilco, Mexico City, Mexico; ^9^Unit of Research, Hospital Juárez de México, Mexico City, Mexico

**Keywords:** systemic lupus erythematosus, interferons lambda, interferon-stimulated genes, oligoadenylate sinthetase-like, rs12979860 SNP

## Abstract

Systemic lupus erythematosus (SLE) is an autoimmune disease with a complex etiology. Various genetic factors are associated with susceptibility to developing SLE and contribute to its onset and progression. Different single-nucleotide polymorphisms (SNPs) have been associated with SLE in several populations. The rs12979860 SNP in interferon lambda 3/4 (IFNλ3/4) is significantly associated with SLE susceptibility in patients negative for nephritis in Taiwanese people, and interferon-stimulated genes (ISGs) are differentially expressed in normal liver by the rs12979860 genotype. This study aimed to investigate whether rs12979860 is associated with the presence of SLE and lupus nephritis in Mexican individuals as well as with the expression of several ISGs in SLE patients. In total, 439 SLE patients and 358 healthy donors were genotyped for rs12979860 using real-time PCR, and allelic discrimination plots were constructed. Additionally, peripheral blood mononuclear cells (PBMCs) were isolated from the venous blood of SLE patients by centrifugation (*n* = 78). The mRNA levels of 2′-5′-oligoadenylate synthetase like (OASL), myxovirus resistance 1 (MX1), 2′5′-oligoadenylate synthetase 1 (OAS1), interferon-stimulated gene 15 (ISG15) and lymphocyte antigen 6 complex, locus E (LY6E) were determined using real-time PCR. The distributions of rs12979860 genotypes and allele frequencies were compared between SLE patients and healthy donors; case-control analysis revealed that rs12979860 was not associated with SLE susceptibility (OR 1.18, 95% CI 0.97–1.45, *p* = 0.08) or with the risk for lupus nephritis (OR 0.913, 95% CI 0.590–1.411, *p* = 0.682). However, OASL expression levels in PBMCs were significantly different between rs12979860 genotypes in SLE patients: median OASL mRNA levels were significantly higher in patients carrying the CC genotype (197.10, IQR 71.10–411.17) than in those with CT/TT genotypes (173.75, IQR 58.80–278.75, *p* = 0.016). Our results suggest that the SNP rs12979860 does not play a relevant role in susceptibility to SLE in Mexican individuals. However, IFNλ3/4 genotypes appear to be associated with OASL expression in PBMCs from patients with SLE.

## Introduction

Systemic lupus erythematosus (SLE) is an inflammatory and autoimmune disorder with a wide range of clinical manifestations that can affect different organs, such as the kidneys, skin, joints, and brain (Tsokos, 2011). Abnormalities in the immune system, circulation of autoantibodies in the body, deposit of immune complexes in tissues, and loss of regulatory function are involved in the pathogenesis of SLE (Zharkova et al., 2017). Various genetics factors are strongly associated with susceptibility to SLE, and several human loci contribute to the pathogenesis and clinical manifestations of SLE (Chung et al., 2011; Bentham et al., 2015). Moreover, some single-nucleotide polymorphisms (SNPs) associated with susceptibility to SLE found within genes are involved in lymphocyte activation, innate immune signaling, and type I interferon (IFN) signaling (Mohan and Putterman, 2015).

Type I IFNs contribute to a breakdown of peripheral tolerance and are important mediators of SLE pathogenesis (Kirou et al., 2005). Type I IFNs can bind to the type I IFN receptor (IFNαR) and thereby induce interferon-stimulated genes (ISGs); moreover, the upregulation of ISGs in peripheral blood is a hallmark of SLE (Baechler et al., 2003; Bennett et al., 2003). Some therapies with biologic agents that target type I IFNs have had mixed efficacy, and it is likely that other signaling pathways play roles in SLE pathogenesis (Borba et al., 2014).

Type III IFN-lambdas (IFNλs), including IFNλ1, IFNλ2, IFNλ3 (also known as IL29, IL28A, and IL28B, respectively), and IFNλ4 (Kotenko et al., 2003; Prokunina-Olsson et al., 2013), interact with specific heterodimeric receptors, such as IFNλR (IFNλR1 and IL10Rβ), and share functional similarities with the type I IFNs (Kotenko et al., 2003). Because IFNλs have immunomodulatory properties in the context of infections and share similar functions with type I IFNs, they may be implicated in autoimmune diseases (Syedbasha and Egli, 2017). Recently, some studies have indicated that SNPs found in IFNλ loci may play a role in the development of lupus nephritis (LN) (Krasnova et al., 2015; Chen et al., 2018). However, the major allele of SNP rs12979860, which is located 3 kb upstream of the IFNλ3 gene, is a significant risk factor for LN in SLE patients, strongly predicts response to IFNα treatment in patients with hepatitis C virus (HCV), and influences ISG mRNA levels and IP-10 serum concentration (Bauer et al., 2009; Urban et al., 2010; Lagging et al., 2011). We previously demonstrated that SNP rs12979860 modulates the *ex vivo* response of peripheral blood mononuclear cells (PBMCs) to IFN-α through the production of IP-10 chemokine in SLE patients (Juárez-Vicuña et al., 2020b).

Despite these observations, the effects of SNP rs12979860 in SLE patients remain somewhat controversial and are not fully understood. Recently, our group investigated that SNP rs12979860 not conferred risk for SLE in a small sample of 164 Mexican patients (Juárez-Vicuña et al., 2020a), for which we evaluated whether this SNP conferred risk for SLE in a larger sample. In this study, we demonstrate that rs12979860 in the IFNλ3/4 locus is not associated with susceptibility to SLE in Mexican patients, although it regulates OASL expression in PBMCs from SLE patients. Our results provide evidence that rs12979860 has important functions in the pathogenesis of SLE.

## Materials and Methods

### Study Participants

In total, 439 patients with SLE were recruited at the rheumatology department of Instituto Nacional de Cardiología Ignacio Chávez, Mexico City, Mexico. All patients fulfilled at least four of the 1997 revised American College of Rheumatology classification (ACR) criteria for SLE (Hochberg, 1997). On enrollment, patients underwent a detailed clinical examination and evaluation of medical charts. Laboratory and radiographic reports were assessed for disease activity and organ-specific damage. The Systemic Lupus Erythematosus Disease Activity Index (SLEDAI-2K) was used to assess disease activity, and patients with scores ≥6 were considered to have active disease (Gladman et al., 2002). We recruited 358 healthy unrelated control individuals without known previous autoimmune diseases. Written informed consent forms were signed by all participants.

### DNA Isolation and Genotyping of SLE Patients and Controls

Fasting venous blood (4 mL) was collected from the patients with SLE and controls using ethylenediaminetetraacetic acid (EDTA-2k)-treated tubes for DNA isolation.

The genomic DNA from patients and controls was extracted from peripheral blood samples using an Ultra Clean Blood Spin DNA Isolation Kit (MoBio Laboratories, United States) following the manufacturer’s instructions. The DNA concentration was determined using a NanoDrop 1000 (Thermo Fisher Scientific, Waltham, MA, United States). Samples with 260/280 ratios ≥1.8 were accepted for genotyping, and the genomic DNA was diluted to 12.5 ng/μL and stored at −20°C until use. The SNP rs12979860 (C/T) was detected by real-time polymerase chain reaction (qPCR) conducted on a LightCycler 480 II (Roche, Rotkreuz, Switzerland) in a total reaction volume of 5.0 μL. The qPCR was performed with 12.5 ng of DNA, 2.5 μL PCR Master Mix (Qiagen, Hilden, Germany), 0.1 μL TaqMan Probes (C_7820464_10 for rs12979860), and 0.4 μL DNase-free water. The allelic discrimination plots for the SNPs in the IFNλ3/4 locus were determined to genotype each sample using LightCycler^®^480 Genotyping Software.

### Blood Samples

PBMCs from 78 patients with SLE were separated using Ficoll-Histopaque-1077 density gradient centrifugation, which were collected in tubes containing EDTA-2k to prevent coagulation (4 mL).

### RNA Extraction

The total RNA was extracted from PBMCs using the TRIzol method (Invitrogen, Carlsbad, CA, United States) according to the manufacturer’s instructions and was frozen at −80°C. The quantity and purity of RNA was determined by absorbance on a NanoDrop 1000 at 260 and 280 nm. Samples with ratios from 1.8 to 2.0 were accepted for the subsequent reverse transcription reaction.

### ISGs Determination by RT-qPCR

The cDNA was synthesized using a Transcriptor First-Strand cDNA Synthesis Kit (Roche, Basel, Switzerland) in a total reaction volume of 15 μL with 160 ng RNA. The reaction was performed as follows: 25°C for 10 min, 42°C for 60 min, and 85°C for 5 min. The expression levels of *OASL* (forward primer 5′-TTGTGCCTGCCTACAGAGC-3′, reverse primer 5′-GATCAGGCTCACATAGACCTCA-3′), *MX1* (forward primer 5′-ACCACAGAGGCTCTCAGCAT-3′, reverse primer 5′-CAGATCAGGCTTCGTCAAGA-3′), *OAS1* (forward primer 5′-GAGAAGGCAGCTCACGAAAC-3′, reverse primer 5′-TCTTAAAGCATGGGTAATTCAGC-3′), *ISG15* (forward primer 5′-GCG AACTCATCTTTGCCAGTA-3′, reverse primer 5′-CCAGCATCTTCACCGTCAGGTC-3′), *LY6E* (forward primer 5′-GCCATCCTCTCCAGAATGAA-3′, reverse primer 5′-GCAGGAGAAGCACATCAGC-3′), and the reference gene *GAPDH* (forward primer 5′-AGCCACATCGCTCAGACAC-3′, reverse primer 5′- GCCCA ATACGACCAAATCC-3′) were determined by qPCR using the LightCycler 480 Probes Master (Applied Biosystems, Foster City, CA, United States) in a LightCycler 480 II thermal cycler (Roche, Rotkreuz, Switzerland). The PCR conditions consisted of an initial denaturation at 95°C for 10 min followed by 40 cycles at 95°C for 15 s, 60°C for 60 s, and 72°C for 1 s. Relative quantification was carried out using the 2^–ΔΔ*Ct*^ method.

### Statistical Methods

The genotype frequencies for SNP rs12979860 in the IFNλ3/4 locus were determined by direct counting, and the allele frequencies were calculated. Finetti^1^ was used to evaluate the Hardy-Weinberg equilibrium (HWE) in the study population. We used a logistic regression adjusted for age and estimated the odds ratio (OR), 95% confidence interval (95% CI), and *P*-value (≤ 0.05) to estimate the effect of rs12979860 in the IFNλ3/4 locus on SLE. The association between allelic and genotypic frequencies was analyzed using IBM SPSS 24 software (Chicago, IL, United States).

The IFN-inducible gene expression was expressed as the median and interquartile range (IQR), comparisons were made using the parametric *t*-test, and comparisons among 3 groups were conducted using one-way ANOVA test (*p* ≤ 0.05). Correlation between groups was evaluated using the Spearman test (*p* ≤ 0.05).

## Results

### Polymorphism rs12979860 C/T in the IFNλ3/4 Locus Is Not Associated With the Risk of SLE in Mexican Patients

In order to investigate the association of IFNλ3/4 locus polymorphisms with SLE susceptibility in Mexican patients, the SNP rs12979860 in the IFNλ3/4 locus was investigated. This study included 439 patients with SLE (mean age of 45.84 ± 16.42 years) and 358 healthy control individuals (mean age of 44.23 ± 15.82 years). Clinical characteristics were stratified by rs12979860 genotype; however, the analysis showed no significant differences between genotypes and clinical characteristics. The SLEDAI-2K score was calculated in all SLE patients, and a SLEDAI-2K score of ≥ 6 was considered as high disease activity (Gladman et al., 2002). At the study visit, recorded disease activity was relatively low with a median SLEDAI- 2K score of 3.

The distribution of all genotypes and alleles of rs12979860 in SLE patients and controls was in Hardy-Weinberg equilibrium (HWE) and is shown in Table 1. The genotype and allelic frequencies of the rs12979860 C/T polymorphism were similar between SLE patients and controls. Moreover, 117 (26.65%) of 439 patients with SLE were homozygous for C/C, 215 (48.97%) were heterozygous, and 107 (24.37%) were homozygous for T/T. In the control group, the genotype distribution was as follows: 111 (31.01%) were homozygous for C/C, 175 (48.88%) were C/T, and 72 (20.11%) were homozygous for T/T.

**TABLE 1:** Genotype and allelic frequencies of rs12979860 C/T polymorphism and association analysis in SLE patients and controls.

SNP	Population	Allele	Genotype (%)						Allele n (%)				
		
		C T	CC	CT	TT	OR	95% CI	*p*	C	T	OR (C vs. T)	95% CI	*p*
rs12979860	Controls (*n* = 358)	**C T**	111 (31.01)	175 (48.88)	72 (20.11)	–	–	–	286 (0.55)	247 (0.45)	–	–	–
	SLE (*n* = 439)		117 (26.65)	215 (48.97)	107 (24.37)	1.19	0.97–1.45	0.08	332 (0.51)	224 (0.49)	1.18	0.97–1.45	0.08

*CI, confidence intervals; OR, odds ratio; SNP, Single Nucleotide Polymorphism; SLE, systemic lupus erythematosus; H-WE, Hardy-Weinberg equilibrium. H-WE p-values to controls (p = 0.84) and SLE (p = 0.67).*

Comparison of rs12979860 C/T alleles indicated that this SNP is not associated with SLE susceptibility in Mexican patients (OR 1.18, 95% IC 0.97–1.45, *p* = 0.08). Similarly, the comparison of rs12979860 C/T between SLE patients and controls did not reveal associations with SLE susceptibility under both the recessive and dominant genetic models (Table 2). Upon applying a dominant genetic model, where the genotypes with the C allele from rs12979860 were grouped, significant differences were not observed (OR 1.28, 95% CI 0.91–1.80, *p* = 0.14). Similarly, the genotypes with the T allele were grouped using a recessive model, which did not show significant differences between CC and CT/TT (OR 1.24, 95% CI 0.91–1.69, *p* = 0.16), indicating that individuals who carry rs12979860 genotypes do not have a genetic risk of SLE.

**TABLE 2:** Association analysis between the rs12979860 C/T SLE patients under different genetic models.

Dominant model						
SNP	Genotypes	Control female	SLE female	OR	95% CI	*p*
	CC/CT	286 (79.9)	332 (75.6)	−	−	−
	TT	72 (20.1)	107 (24.4)	1.28	0.91- 1.80	0.14
	
**rs12979860**	**Recessive model**					
	
	CC	111 (31.0)	117 (26.7)	−	−	−
	CT/TT	247 (69.0)	322 (73.3)	1.24	0.91–1.69	0.16

*CI, confidence intervals; OR, odds ratio; SNP, Single nucleotide polymorphism; SLE, systemic lupus erythematosus.*

We further investigated whether rs12979860 was associated with history of kidney damage (data obtained from medical records) among SLE patients (*n* = 164). The kidney damage history group (*n* = 90) comprised 14 male and 76 female patients with a mean age of 37 ± 12.51 years and median SLEDAI-2K score of 4. The no kidney damage history group (*n* = 74) included 6 male and 68 female patients with a mean age of 41.8 years and median SLEDAI-2K score of 0 (Supplementary Table 1).

As shown in Table 3, alleles of rs12979860 are not significantly associated with a history of kidney damage in SLE patients (OR 0.913, 95% CI 0.590–1.411, *p* = 0.682). Furthermore, we compared the genotypes of rs12979860 C/T between SLE patients with kidney damage history and those without kidney damage history under the genetic models (Table 4). Upon applying a dominant genetic model, significant differences were not found (OR 0.906, 95% CI 0.433–1.896, *p* = 0.794). Similarly, comparison of rs12979860 C/T between SLE patients with a history of kidney damage under a recessive model did not show an association with prior kidney damage (OR 0.866, 95% CI 0.433–1.732, *p* = 0.685, Table 4).

**TABLE 3:** Genotype and allelic frequencies of rs12979860 C/T polymorphism and association analysis in SLE patients; no kidney damage history vs. kidney damage history.

SNP		Kidney damage history (*n* = 90)	No kidney damage history (*n* = 74)	OR	95% CI	*p*
	Genotype					
	CC	23 (25.6)	21 (28.4)	*−*	*−*	*−*
	CT	46 (51.1)	37 (50.0)	0.881	0.423–1.834	0.735
**rs12979860**	TT	21 (23.3)	16 (21.6)	0.834	0.346–2.010	0.687
	Allele					
	C	92	79	*−*	*−*	*−*
	T	88	69	0.913	0.590–1.411	0.682

*CI, confidence intervals; OR, odds ratio; SNP, Single Nucleotide Polymorphism; SLE, systemic lupus erythematosus.*

**TABLE 4:** Association analysis between the rs12979860 C/T SLE patients no kidney damage history vs. kidney damage history under different genetic models.

Dominant model						
SNP	Genotypes	Kidney damage history (*n* = 90)	No kidney damage history (*n* = 74)	OR	95% CI	*p*
	CC/CT	69 (76.7)	58 (78.4)	−	−	−
	TT	21 (23.3)	16 (21.6)	0.906	0.433–1.896	0.794
	
**rs12979860**	**Recessive model**					
	
	CC	23 (25.6)	21 (28.4)	−	−	−
	CT/TT	67 (74.4)	53 (71.6)	0.866	0.433–1.732	0.685

*CI, confidence intervals; OR, odds ratio; SNP, Single Nucleotide Polymorphism; SLE, systemic lupus erythematosus.*

### The rs12979860 Genotypes Affect OASL Expression in PBMCs From SLE Patients

**FIGURE 1:**
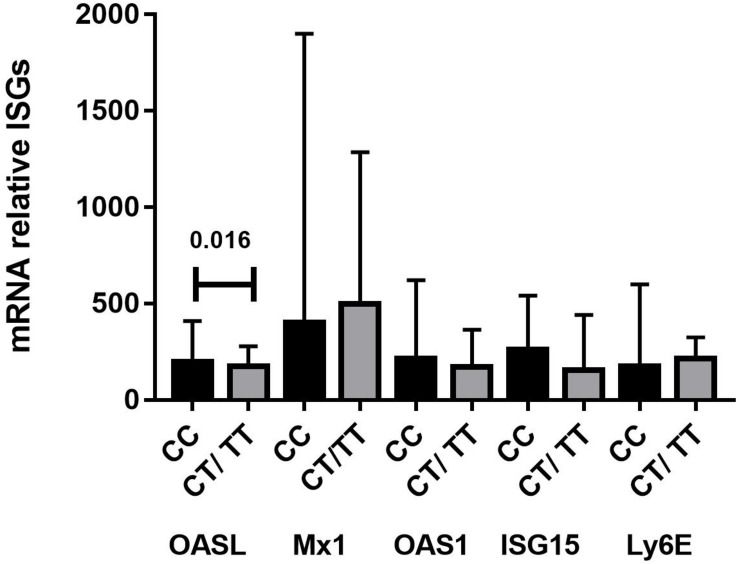
Levels of ISGs mRNA in PBMC from SLE patients grouped according to their IFNλ3 and IFNλ4 genotype combinations (*n* = 78). RT-qPCR was used to determine the relative expression level of OASL, MX1, OAS1, ISG15, and LY6E RNA in the PBMCs of SLE. Significantly higher mRNA levels of OASL were measured in SLE patients that are carriers of the IFNλ3/4 rs12979860 CC genotype vs. CT/TT genotypes. The data were analyzed using Student’s *t*-test and results are expressed as ratio to GAPDH (median, interquartile range), before analysis the values obtained of 2^–ΔΔ*Ct*^ for mRNA OASL, MX1, OAS1, LY6E were multiplied by 1,000 and the values of 2^–ΔΔ*Ct*^ for ISG15 were multiplied by 100,000.

We next investigated whether rs12979860 genotypes influenced ISG expression in PBMCs from SLE patients. We quantified the gene expression of OASL, MX1, OAS1, ISG15, and LY6E in PBMCs from 78 SLE patient samples. Interestingly, we found a statistically significant association between rs12979860 genotype variants and the mRNA expression levels of OASL, as SLE patients carrying the CC genotype (median 197.10, IQR 71.10–411.17) showed significantly higher median OASL mRNA levels than patients with CT or TT genotypes (median: 172.80, IQR 47.70–294.00 vs. 174.70, IQR 61.95–239.65, respectively, *p* = 0.050, Table 5). Specifically, the most prominent difference was observed in the recessive genetic model between the CC and CT/TT groups (median: 197.10, IQR 71.10–411.17 vs. 173.75, IQR 58.80–278.75, respectively, *p* = 0.016, Figure 1). Additionally, the rs12979860 genotype did not influence others ISGs levels in PBMCs from SLE patients; we only observed a trend in which SLE patients carrying the CC genotype showed high expression of ISGs compared to that of patients with CT or TT genotypes (Figure 1).

**TABLE 5:** The relationship between the rs12979860 C/T and expression levels of OASL, MX1, OAS1, ISG15, and LY6E in PBMCs from SLE patients.

	rs12979860			
ISG	CC	CT	TT	*p*
OASL	197.10 (71.10–411.17)	172.80 (47.70–294.00)	174.70 (61.95–239.65)	0.050*
MX1	401.70 (161.40–1901.00)	492.70 (257.00–1102.00)	560.65 (334.60–1439.00)	0.666
OAS1	215.50 (82.00–623.10)	229.50 (94.10–344.80)	172.10 (124.20–490.20)	0.355
ISG15	0.26 (0.08–0.54)	0.13 (0.07–0.43)	0.20 (0.10–0.53)	0.368
LY6E	175.60 (91.30–601.60)	199.60 (68.40–311.10)	218.95 (124.17–327.35)	0.281

ISG, IFN-stimulated gene; OASL, OAS-like protein; MX1, myxovirus resistance protein 1; OAS1, 2′–5′-oligoadenylate synthetase 1; ISG15, Interferon-stimulated gene 15; LY6E, lymphocyte antigen 6 complex locus E; SLE, systemic lupus erythematosus; *p statistically significant (≤ 0.05). The dates are expressed as median (Interquartile range) and data were analyzed by ANOVA test.

### Correlation Between ISG mRNA Levels and Disease Activity Index in SLE Patients

We found that the mRNA levels of OASL (median: 126.35, IQR 61.87–308.20 vs. 208.50, IQR 158.70–479.22, *p* = 0.039), MX1 (median: 450.10, IQR 251.20–1296.00 vs. 980.20, IQR 335.70–2797.50, *p* = 0.007), and LY6E (median: 178.40, IQR 78.40–332.65 vs. 273.60, IQR 145.30–576.70, *p* = 0.050) in active SLE patients were significantly higher than those in patients with inactive SLE. Among SLE patients, mRNA levels of OAS1 (median 171.65, IQR 103.52–364.12 vs. 269.80, IQR 90.20–1063.60, *p* = 0.985) and ISG15 (median 0.15, IQR 0.07–0.46 vs. 0.27, IQR 0.14–0.53, *p* = 0.355) were slightly higher in those who had active disease than in those who did not; however, the differences were not statistically significant.

We further investigated the correlation between ISG mRNA levels and SLEDAI-2K score. The correlation analysis showed a significant positive correlation between the mRNA levels of OASL (*r* = 0.259, *p* = 0.030), MX1 (*r* = 0.255, *p* = 0.029), ISG15 (*r* = 0.242, *p* = 0.046), and LY6E (*r* = 0.250, *p* = 0.032) and SLEDAI-2K score in SLE patients. Similarly, mRNA levels of OASL (*r* = −0.461, *p* < 0.001), MX1 (*r* = −0.420, *p* < 0.001), OAS1 (*r* = −0.333, *p* = 0.002), ISG15 (*r* = −0.529, *p* < 0.001), and LY6E (*r* = −0.464, *p* < 0.001) negatively correlate with serum complement C3 levels. The correlation analysis showed a significant negative correlation between mRNA levels of OASL (*r* = −0.537, *p* < 0.001), MX1 (*r* = −0.544, *p* < 0.001), OAS1 (*r* = −0.442, *p* < 0.001), ISG15 (*r* = −0.577, *p* < 0.001), and LY6E (*r* = −0.501, *p* < 0.001) and C4 levels.

In addition, the SLE cohort was segregated into patients with presence or absence of clinical manifestations (musculoskeletal, cardiopulmonary, hematologic abnormalities and renal involvement) according to medical records, and expression ISGs mRNA levels were not different between groups (*p* > 0.05, Supplementary Table 2).

Furthermore, we analyzed expression levels between the serological features of SLE. We found that the mRNA levels of OASL (median: 243.95, IQR 127.07–375.87 vs. 112.90, IQR 49.30–219.45, *p* = 0.002), MX1 (median: 859.20, IQR 369.92–1844.50 vs. 367.50, IQR 163.75–1172.00, *p* = 0.004), ISG15 (median: 0.31, IQR 0.13–0.59 vs. 0.13, IQR 0.06–0.32, *p* = 0.001) and LY6E (median: 258.30, IQR 164.77– 398.92 vs. 156.80, IQR 67.80–452.70, *p* = 0.05) in SLE with low C3 levels were significantly higher than those in patients with normal levels of C3 (Supplementary Table 3).

Interestingly, mRNA levels of OASL (median: 338.7, IQR 204.80–534.85 vs. 116.90, IQR 51.50–228.00, *p* < 0.001), MX1 (median: 1304.00, IQR 638.80–2679.50 vs. 375.70, IQR 214.10–1071.00, *p* < 0.001), OAS1 (median: 308.20, IQR 174.15–1027.10 vs. 168.00, IQR 94.10–335.30, *p* = 0.004), ISG15 (median: 0.51, IQR 0.32–0.64 vs. 0.13, IQR 0.07–0.30) and LY6E (median: 378.60, IQR 299.50–632.25 vs. 16.80, IQR 74.40–235.90, *p* < 0.001) in SLE patients with low C4 levels were significantly higher than in those who did not (Supplementary Table 3). We further compared the mRNA levels ISGs between SLE patients with low CRP (C-reactive protein) levels and patients with normal levels of CRP, we did not significative differences between groups (*p* > 0.05, Supplementary Table 3).

Finally, we used autoantibody (anti-DNA, anti-Ro/SSA, anti-La/SSB, anti-Sm (Smith), and anti-Sm/RNP) for sub-classification of SLE patients. SLE patients were segregated into autoantibody (+) and (−). The mRNA levels of OASL (median: 138.20, IQR 46.05–289.42 vs. 224.20, IQR 99.27–369.82, *p* = 0.047), OAS1 (median: 144.65, IQR 82.97–234.20 vs. 262.00, IQR 120.37–778.12, *p* = 0.013), ISG15 (median: 0.14, IQR 0.07–0.26 vs. 0.31, IQR 0.12–0.51, *p* = 0.044) and LY6E (median: 147.05, IQR 65.05–235.92 vs. 317.60, IQR 161.35–385.37, *p* = 0.030) in SLE patients that were anti-DNA (−) were significantly lower than those in patients with anti-DNA (+). Among SLE patients, mRNA levels of MX1 (median: 453.40, IQR 200.85–973.45 vs. 968, IQR 284.90–2380.75) were slightly lower in those who were anti-DNA (−) than in those who did not: however, the differences were not significant (Supplementary Table 4). ISGs mRNA levels were not different in the other autoantibodies (*p* > 0.05, Supplementary Table 4).

## Discussion

In this study, we reported that the SNP rs12979860 is not associated with susceptibility to SLE in Mexican patients, although its genotypes correlate with differential OASL expression in PBMCs. We previously reported that SNP rs12979860 is not associated with SLE in a small sample, interestingly when increase the sample size the result duplicate (Juárez-Vicuña et al., 2020a). The SNP rs12979860 resides in the IFNλ3/4 gene, and its functional influence on SLE remains to be identified. There are few studies on the association between IFNλ gene polymorphisms and SLE. It was determined that the minor alleles of rs12979860 T tended to associate with SLE in Taiwanese individuals, and the major allele of rs12979860 C is a major risk factor for nephritis in SLE patients (Chen et al., 2018). Other studies have reported that alleles from rs12979860 are linked to increased nephritis severity, the activity of a renal lupus process, and the rate of SLE and LN onset (Krasnova et al., 2015). Our results are inconsistent, and this discrepancy might be explained by studies with inadequate statistical power due to limited sample size and racial and ethnic differences.

IFNλs are important cytokines in antiviral immune responses; however, it has been suggested that IFNλs are involved in SLE pathogenesis (Chyuan et al., 2019). IFNλs promote immune dysregulation and tissue inflammation in TLR7-induced lupus and regulate tissue inflammation through specifically affecting skin and kidney cells (Goel et al., 2020). Patients with SLE have increased serum IFN-λ1 protein levels, and the highest levels are positively correlated with disease activity as well as with anti-dsDNA antibody and C-reactive protein (Wu et al., 2011). Approximately 40–70% of SLE patients develop LN, and many investigators have hypothesized that IFNλ plays a pathogenic role in LN; moreover, IFNλ is expressed in renal biopsies from patients with LN, its expression is most pronounced in glomeruli, and it is found in inflammatory infiltrates of CD3 + T cells and tubular cells (Zickert et al., 2016).

Furthermore, we examined whether rs12979860 could regulate ISG expression in SLE patients. Interestingly, our results showed that among ISGs, only the expression of OASL and rs12979860 genotypes are linked to SLE patients. Previously, it was reported that genotype CC of rs12979860 was associated with higher expression of some ISGs in normal liver, including IFI44, RSAD2, ISG15, IFI27, LGALS3BP, OAS3, IFI6, HTATIP2, and IFIH1 (Raglow et al., 2013). OASL expression is regulated by some SNPs that cause decreased OASL levels (Su et al., 2008).

Our result indicated that mRNA levels of ISGs are correlated with active SLEDAI-2K scores and associated with active SLE. Several studies have confirmed that ISG mRNA levels are correlated with disease activity and play a role in SLE pathogenesis (Feng et al., 2015). Additionally, it has been suggested that ISGs could be used as predictive biomarkers to identify patients with a favorable response to anti-type I IFN therapy (Yao et al., 2010).

OASL is related to the OAS proteins by its N-terminal OAS-like domain, belongs to an ISG family, lacks 2′–5′ oligoadenylate synthetase activity, and is induced by pathway interferon regulatory factor (IRF)-3 as well as by IFN signaling (Zhu et al., 2015). Expression of OASL is upregulated in some diseases, including SLE, systemic sclerosis, and juvenile dermatomyositis (Ye et al., 2007; de Freitas Almeida et al., 2014; Musumeci et al., 2018). In light of this, OASL expression can be useful as a biomarker of SLE, as relative OASL mRNA expression levels are upregulated in active SLE patients and OASL may be a discriminant for systemic infection in SLE (Ye et al., 2007). However, patients with active SLE who had renal disorders showed upregulated OASL in PBMCs and CD19^+^ B cells compared to that in patients without clinical manifestations (Gao et al., 2020).

The exact mechanism by which rs12979860 regulates expression levels of OASL remains unknown, but we performed meta-transcriptomic analysis using RNA-seq dataset provided by the Genotype-Tissue Expression (GTEx) Project (Lonsdale et al., 2013) and found that IFN-λ4 (Interferon lambda 4), PAK4 [p21 (RAC1) activated kinase 4], and NCCRP1 (F-box associated domain containing) genes in cis are regulated by rs12979860 (*p* < 0.001). IFN-λ4 gene is also regulated by rs368234815−TT/ΔG SNP (Prokunina-Olsson et al., 2013) that is in strong linkage disequilibrium with rs12979860 and both SNP regulate extrahepatic ISGs expression in chronic HCV patients including OASL (Rosenberg et al., 2018). The frequency allelic of rs12979860 and rs368234815 polymorphisms are similar between individuals from different regions of Mexico (Gonzalez-Aldaco et al., 2016).

IFN-λ and IFN-α induce expression of ISGs through activating the JAK-STAT signaling pathway. The regulation of IFNLR expression may greatly affect binding to IFN-λs and accordingly influence the JAK-STAT signaling pathway and expression of ISGs (Syedbasha and Egli, 2017). There is no evidence to suggest that rs12979860 genotypes modulate IFNLR expression levels; however, it was recently reported that rs12979860 upregulated IFNAR1 expression in PBMCs from healthy donors in a time- and dose-dependent manner (Lalle et al., 2014). Among the identified SNPs, rs10903035, located within the 3′-UTR of the IFNLR1 gene, was found to be most relevant and may modulate gene expression and function (Jiménez-Sousa et al., 2013).

Our study has some limitations. Although we found that rs1297960 SNP is not associated with susceptibility to SLE in Mexican patients and could regulate OASL expression, the exact mechanism remains unknown. Given these conflicting results, additionally mechanistic studies are necessary to investigate the role of rs12979860 in SLE. In summary, our results suggest that rs12979860 does not influence susceptibility to SLE in Mexican individuals, although rs12979860 genotypes appear to regulate OASL expression in PBMCs from patients with SLE.

## Data Availability Statement

The raw data supporting the conclusions of this article will be made available by the authors, without undue reservation.

## Ethics Statement

The studies involving human participants were reviewed and approved by the Comisión de Bioética del Instituto Nacional de Cardiología Ignacio Chávez (No. 12–771). The patients/participants provided their written informed consent to participate in this study.

## Author Contributions

YJ-V and FS-M performed experiments and drafted the manuscript. JP-R, LA-P, FS, LM-M, AH-D, and JR-B supervised the work and analyzed data. YJ-V, MO-S, and EP-O performed the analyzed the data and performed the laboratory experiments. LA-G and FS-M designed the research, supervised the work and provided the reagents. JR-B and AH-D performed the bioinformatics analyzed. LM-M and LA-G performed and analyzed the clinical data. All authors listed have made a substantial, direct and intellectual contribution to the work, and approved it for publication.

## Conflict of Interest

The authors declare that the research was conducted in the absence of any commercial or financial relationships that could be construed as a potential conflict of interest. The handling editor declared a past co-authorship with the authors JR-B and FS-M.

## Footnotes

1 http://ihg.gsf.de/cgi-bin/hw/hwa1.pl
