# Supplementary material for: Interferon Lambda 3/4 (IFNλ3/4) rs12979860 Polymorphisms Is Not Associated With Susceptibility to Systemic Lupus Erythematosus, Although It Regulates OASL Expression in Patients With SLE
Source: Front Genet. 2021 Jun 2;12:647487. doi: 10.3389/fgene.2021.647487 (PMC8206639; doi:10.3389/fgene.2021.647487)
Supplement: Supplementary file 2 [file Data_Sheet_2.docx]

**Supplementary table 1.** Clinical characteristics and laboratory parameters of SLE patients with kidney damage history and non-kidney damage history

|  | **SLE patients with kidney damage**  **(n= 90)** | **SLE patients non-kidney damage history**  **(n=74)** |
| --- | --- | --- |
| Age years | 37.0 ± 12.51 | 41.80 ± 13.33 |
| Male/female | 14/76 | 6/68 |
| SLEDAI-2K score, median (IQR) | 4 (0-6) | 0 (0-4) |
| C3, mg/L | 87.66± 26.19 | 99.54± 23.06 |
| C4, mg/L | 15.29± 8.36 | 16.11± 7.73 |
| CRP mg/L | 13.93± 46.21 | 5.00± 12.25 |
| ANA (+) | 77 (85.5%) | 63 (85.1%) |
| Anti-dsDNA, + (%) | 39 (43.3%) | 19 (25.6%) |
| Anti-Sm (Smith), + (%) | 27 (30%) | 10 (13.5%) |
| Anti-Sm/ RNP, + (%) | 28 (31.1%) | 18 (24.3%) |
| Anti-Ro/ SSA, + (%) | 39 (43.3%) | 18 (24.3%) |
| Anti-La/ SSB, + (%) | 12 (13.3%) | 10 (13.5%) |
| Anticardiolipin IgG, + (%) | 12 (13.3%) | 10 (13.5%) |
| Anticardiolipin IgM, + (%) | 5 (5.5%) | 8 (10.8%) |

Data are reported as median± SD unless otherwise specified. ANA: antinuclear antibody; C3: complement 3; C4: complement 4; CRP: C-reactive protein; ds-DNA: anti-double stranded DNA antibody; SD: standard deviation; SLE: systemic lupus erythematosus; SLEDAI-2K: SLE disease activity index; Smith, anti-smith-antibody.

**Supplementary table 2.** mRNA levels of OASL, MX1, OAS1, ISG15 and LYE6 expression by presence or absence of SLE clinical features.

| **Features** | **ISGs** | **Present** | **Absent** | **P value** |
| --- | --- | --- | --- | --- |
| Musculoskeletal and cutaneous involvement | OASL | 113.10  (37.17- 327.92) | 180.60  (68.90- 315.70) | 0.409 |
|  | MX1 | 486.40  (190.42- 1677.00) | 511.05  (274.10- 1313.25) | 0.817 |
|  | OAS1 | 135.30  (89.95- 811.37) | 228.70  (108.15- 436.17) | 0.815 |
|  | ISG15 | 0.39  (0.03- 0.54) | 0.18  (0.08-0.46) | 0.802 |
|  | LY6E | 201.80  (95.95- 423.70) | 197.55  (90.95- 423.70) | 0.915 |
| Cardiopulmonary | OASL | 212.30  (80.02- 355.70) | 167.40  (71.10- 308.20) | 0.760 |
|  | MX1 | 1019.10  (220.77- 1270.00) | 468.40  (254.10- 1313.25) | 0.795 |
|  | OAS1 | 201.00  (141.72- 348.17) | 200.00  (97.32- 449.55) | 0.775 |
|  | ISG15 | 0.23  (0.09- 0.83) | 0.18  (0.07- 0.48) | 0.531 |
|  | LY6E | 215.35  (81.12- 321.30) | 194.10  (90.95- 336.40) | 0.727 |
| Hematologic abnormalities | OASL | 247.30  (200.50- 337.47) | 159.80  (63.97- 315.70) | 0.120 |
|  | MX1 | 1135.00  (355.30- 2214.00) | 468.40  (248.30- 1299.75) | 0.337 |
|  | OAS1 | 264.40  (109.52- 5968.50) | 200.20  (98.80- 419.45) | 0.699 |
|  | ISG15 | 0.48  (0.29- 0.59) | 0.15  (0.07- 0.43) | 0.033* |
|  | LY6E | 247.50  (202.37- 520.22) | 186.50  (78.20- 333.50) | 0.248 |
| Renal  involvement | OASL | 190.90  (38.20 381.00) | 174.70  (75.00- 373.60) | 0.776 |
|  | MX1 | 346.90  (200.10- 1933.00) | 492.70  (280.70- 1304.00) | 0.788 |
|  | OAS1 | 227.90  (98.40-308.20) | 172.50  (100.00- 441.20) | 0.767 |
|  | ISG15 | 0.14  (0.05- 0.44) | 0.19  (0.08- 0.50) | 0.750 |
|  | LY6E | 192.70  (62.20- 354.30) | 207.70 (107.20- 335.30) | 0.736 |

Data was expressed as median (interquartile range) and comparisons were made using the nonparametric Mann–Whitney test (p<0.05). Clinical manifestations were defined as follows: musculoskeletal and cutaneous involvement: ≥2 joints with pain and swelling or effusion confirmed by physical examination; and/or proximal muscle weakness associated with elevated creatine phosphokinase/aldolase; and/or malar rash, patchy or diffuse alopecia, oral or nasal ulceration, photosensitivity or discoid rash.

Cardiopulmonary involvement: pleural or pericardial rubbing and effusion confirmed by chest X-ray or echocardiography, respectively, and/or ulceration, gangrene, evidence of periungueal infarction or splinter hemorrhages on the dermatoscopic examination using non-contact, polarized light DermLite Photo dermatoscope (3Gen, San Juan Capistrano, CA, USA). Hematological involvement: platelet count <100,000/mL, white blood cell count <3,000/mL, or autoimmune hemoytic anemia.

Renal involvement: ≥0.5 g/24 h urine proteins, <50% glomerular filtration rate, >5 white or red blood cells/high-power field of unspun urine, or urinary casts.

Neuropsychiatric involvement: seizures, psychosis not associated with the use of glucocorticoids, acute confusional state, lupus migraine, cranial neuropathy, cognitive impairment, or transverse myelitis.

**Supplementary table 3.** mRNA levels of OASL, MX1, OAS1, ISG15 and LYE6 expression by low and normal levels of serological features.

| **ISG** | **C3** **(0- 89.9 mg/dL)** | **C3 (> 90 mg/dL)** | **p value** |
| --- | --- | --- | --- |
| OASL | 243.95 (127.07- 375.87) | 112.90 (49.30- 219.45) | 0.002* |
| MX1 | 859.20 (369.92- 1844.50) | 367.50 (163.75- 1172.00) | 0.004* |
| OAS1 | 253.40 (126.22- 384.57) | 169.70 (85.55- 457.90) | 0.127 |
| ISG15 | 0.31 (0.13- 0.59) | 0.13 (0.06- 0.32) | 0.001* |
| LY6E | 285.30 (164.77- 398.92) | 156.80 (67.80- 452.70) | 0.005* |
|  | **C4 (0- 9.9 mg/dL)** | **C4 (> 10 mg/dL)** | **p value** |
| OASL | 338.70 (204.80- 534.85) | 116.90 (51.50- 228.00) | <0.001* |
| MX1 | 1304.00 (638.80-2679.50) | 375.70 (214.10-1071.00) | <0.001* |
| OAS1 | 308.20 (174.15- 1027.10) | 168.00 (94.10- 335.30) | 0.004* |
| ISG15 | 0.51(0.32- 0.64) | 0.13 (0.07- 0.30) | <0.001* |
| LY6E | 378.60 (299.50- 632.25) | 163.80 (74.40- 235.90) | <0.001* |
|  | **CRP (0-5 mg/L)** | **CRP (>5.1 mg/L)** | **p value** |
| OASL | 192.55 (87.85- 315.70) | 104.50 (49.60- 271.35) | 0.178 |
| MX1 | 660.95 (262.92- 1327.75) | 368.00 (239.95- 1187.50) | 0.203 |
| OAS1 | 200.20 (112.50- 422.87) | 172.10 (88.05- 476.80) | 0.530 |
| ISG15 | 0.20 (0.08- 0.49) | 0.13 (0.07- 0.43) | 0.519 |
| LY6E | 211.60 (104.65- 338.60) | 167.70 (69.00- 305.40) | 0.256 |

Data was expressed as median (interquartile range) and comparisons were made using the nonparametric Mann-Whitney test (p< 0.05). Laboratories normal range for C3 is 90–180 mg/dl, for C4 is 10–40 mg/dl and CRP is 0.0–5.0 mg/l. C3: complement 3; C4: complement 4; CRP: C-reactive protein

**Supplementary table 4.** mRNA levels of OASL, MX1, OAS1, ISG15 and LYE6 expression by autoantibodies (+) and autoantibodies (−).

| **ISG** | **Anti-DNA (-)** | **Anti-DNA (+)** | **p value** |
| --- | --- | --- | --- |
| OASL | 138.20 (46.05- 289.42) | 224.20 (99.27- 369.82) | 0.047* |
| MX1 | 453.40 (200.85- 973.45) | 968.20 (284.90- 2380.75) | 0.114 |
| OAS1 | 144.65 (82.97- 234.20) | 262.00 (120.37- 778.12) | 0.013* |
| ISG15 | 0.14 (0.07- 0.26) | 0.31 (0.12- 0.51) | 0.044* |
| LY6E | 147.05 (65.05- 235.92) | 317.60 (161.35- 385.37) | 0.030* |
|  | **Anti-Ro/SSA (-)** | **Anti-Ro/SSA (+)** | **p value** |
| OASL | 118.80 (36.70- 298.75) | 220.25 (87.35- 361.35) | 0.093 |
| MX1 | 456.70 (207.60- 1618.00) | 496.85 (235.25- 1448.75) | 0.823 |
| OAS1 | 144.80 (96.25- 361.65) | 244.15 (121.65- 477.87) | 0.244 |
| ISG15 | 0.15 (0.06- 0.32) | 0.28 (0.11- 0.55) | 0.050 |
| LY6E | 175.60 (68.40- 317.60) | 229.45 (131.55- 398.92) | 0.148 |
|  | **Anti-La/SSB (-)** | **Anti-La/SSB (+)** | **p value** |
| OASL | 157.60 (41.10- 303.50) | 175.20 (71.00- 343.05) | 0.393 |
| MX1 | 456.70 (201.10- 1287.00) | 677.00 (273.42- 1908.75) | 0.185 |
| OAS1 | 144.40 (90.70- 299.30) | 332.10 (223.02- 668.62) | 0.009* |
| ISG15 | 0.19 (0.07- 0.41) | 0.24 (0.09- 0.49) | 0.272 |
| LY6E | 156.80 (66.00- 311.10) | 247.65 (191.70- 477.80) | 0.091 |
|  | **Anti-Sm/RNP (-)** | **Anti-Sm/RNP (+)** | **p value** |
| OASL | 154.20 (40.37- 369.82) | 222.75 (105.45- 312.95) | 0.633 |
| MX1 | 367.75 (190.42- 2020.25) | 679.05 (300.95- 1327.25) | 0.420 |
| OAS1 | 228.70 (97.32- 459.00) | 171.45 (103.95- 298.60) | 0.886 |
| ISG15 | 0.16 (0.06- 0.32) | 0.36 (0.15- 0.53) | 0.045* |
| LY6E | 166.20 (65.05- 363.37) | 229.30 (172.37- 333.50) | 0.078 |
|  | **Anti-Sm (Smith) (-)** | **Anti-Sm (Smith) (-)** | **p value** |
| OASL | 196.60 (62.70- 367.20) | 204.80 (87.32- 294.95) | 0.720 |
| MX1 | 480.10 (201.10- 1901.00) | 1014.60 (279.20- 1832.25) | 0.453 |
| OAS1 | 168.00 (100.00- 434.50) | 244.50 (68.27- 482.35) | 0.778 |
| ISG15 | 0.19 (0.09- 0.51) | 0.29 (0.08- 0.48) | 0.948 |
| LY6E | 186.40 (103.80- 378.60) | 252.05 (145.47- 352.90) | 0.541 |

Data was expressed as median (interquartile range) and comparisons were made using the nonparametric Mann-Whitney test (p< 0.05).
